# Isolation of a Highly Purified HSC-enriched CD34^+^CD90^+^CD45RA^−^ Cell Subset for Allogeneic Transplantation in the Nonhuman Primate Large-animal Model

**DOI:** 10.1097/TXD.0000000000001029

**Published:** 2020-07-15

**Authors:** Stefan Radtke, Lucrezia Colonna, Anai M. Perez, Michelle Hoffman, Leslie S. Kean, Hans-Peter Kiem

**Affiliations:** 1 Stem Cell and Gene Therapy Program, Fred Hutchinson Cancer Research Center, Seattle, WA.; 2 Ben Towne Center for Childhood Cancer Research, Seattle Children’s Research Institute, Seattle, WA.; 3 Department of Pediatrics, University of Washington, Seattle, WA.; 4 Department of Medicine, University of Washington, Seattle, WA.; 5 Department of Pathology, University of Washington School of Medicine, Seattle, WA.

## Abstract

Allogeneic hematopoietic stem cell transplantation (allo-HCT) is a common treatment for patients suffering from different hematological disorders. Allo-HCT in combination with hematopoietic stem cell (HSC) gene therapy is considered a promising treatment option for millions of patients with HIV+ and acute myeloid leukemia. Most currently available HSC gene therapy approaches target CD34-enriched cell fractions, a heterogeneous mix of mostly progenitor cells and only very few HSCs with long-term multilineage engraftment potential. As a consequence, gene therapy approaches are currently limited in their HSC targeting efficiency, very expensive consuming huge quantities of modifying reagents, and can lead to unwanted side effects in nontarget cells. We have previously shown that purified CD34^+^CD90^+^CD45RA^−^ cells are enriched for multipotent HSCs with long-term multilineage engraftment potential, which can reconstitute the entire hematopoietic system in an autologous nonhuman primate transplant model. Here, we tested the feasibility of transplantation with purified CD34^+^CD90^+^CD45RA^−^ cells in the allogeneic setting in a nonhuman primate model.

**Methods.:**

To evaluate the feasibility of this approach, CD34^+^CD90^+^CD45RA^−^ cells from 2 fully major histocompatibility complex-matched, full sibling rhesus macaques were sort-purified, quality controlled, and transplanted. Engraftment and donor chimerism were evaluated in the peripheral blood and bone marrow of both animals.

**Results.:**

Despite limited survival due to infectious complications, we show that the large-scale sort-purification and transplantation of CD34^+^CD90^+^CD45RA^−^ cells is technically feasible and leads to rapid engraftment of cells in bone marrow in the allogeneic setting and absence of cotransferred T cells.

**Conclusions.:**

We show that purification of an HSC-enriched CD34^+^ subset can serve as a potential stem cell source for allo-HCTs. Most importantly, the combination of allo-HCT and HSC gene therapy has the potential to treat a wide array of hematologic and nonhematologic disorders.

Allogeneic hematopoietic cell transplantation (allo-HCT) is a promising curative treatment strategy for an increasing number of malignant and nonmalignant hematological diseases, including different types of leukemia, thalassemia, and autoimmune disorders.^1,2^ Furthermore, allo-HCT is considered a potential treatment option for patients with HIV who develop secondary hematologic malignancies, by employing donors who bear an inactivating mutation in the coreceptor CCR5 that confers natural resistance to HIV infection.^3–5^ Since HIV-resistant donors are rare, a combination of allo-HCT with hematopoietic stem cell (HSC) gene therapy targeting the CCR5 receptor in donor HSC to render them HIV-resistant has been discussed as an alternative strategy.^6–8^ In addition, patients affected by acute myeloid leukemia could benefit from a combination of allo-HSC transplantation and gene therapy, via the editing of the myeloid marker CD33 in donor HSCs, in order to confer resistance to anti-CD33 targeted chemotherapy.^9–11^

Novel approaches aiming to combine allo-HCT with HSC gene therapy/editing involve technical and financial difficulties. All currently existing gene therapy/editing approaches target CD34^+^ cells, which are a heterogenous mix mostly containing short-term progenitor cells and <0.1% HSCs with long-term engraftment potential.^12^ The inability to purify and specifically target multipotent HSCs limits the targeting efficiency,^7,13–15^ increases the costs for modifying reagents,^16–18^ and poses the risk of potential gene therapy off-target effects.^19–25^

CD34^+^ hematopoietic stem and progenitor cells (HSPCs) can be subdivided into 3 different subsets based on the expression of the cell surface markers CD90 and CD45RA. Additional assessment of these markers allows to distinguish 3 CD34 subsets enriched for HSCs (CD90^+^CD45RA^−^), multipotent and erythro-myeloid progenitors (CD90^−^CD45RA^−^), and lympho-myeloid progenitors (CD90^−^CD45RA^+^).^26^ By performing competitive reconstitution experiments, we have recently described that CD34^+^CD90^+^CD45RA^−^ cells represent the 1 subset to be exclusively required for rapid hematopoietic recovery, robust long-term multilineage engraftment, and for the entire reconstitution of the bone marrow (BM) stem cell compartment in both an autologous nonhuman primate (NHP) stem cell transplantation and gene therapy model^26^ and in an HSC xenograft murine model.^27^ Most importantly, this HSC-enriched phenotype is evolutionarily conserved between humans and NHPs^26^ and reduces the number of target cells necessary for gene therapy/editing up to 20-fold.^28^ However, to date, transplantation with purified CD34^+^CD90^+^CD45RA^−^ HSCs has not been tested in allogeneic setting, wherein these cells could potentially represent a major advance by making gene-edited allo-HCT more efficient and successful.

Here, we hypothesized that allogeneic transplantation of HSC-enriched CD34^+^CD90^+^CD45RA^−^ would result in multilineage reconstitution in the BM and significantly reduce the target cells number for the development of combined allo-HCT gene therapy approaches. For this purpose, 2 major histocompatibility complex (MHC)-matched, full sibling rhesus macaques were transplanted with sort-purified CD34^+^CD90^+^CD45RA^−^ cells, and donor chimerism evaluated in the peripheral blood (PB) and BM. Despite early termination of the study because of infectious complications, we observed engrafted CD34^+^ HSPCs, rapid onset of donor chimerism in the BM, and onset of donor chimerism in the PB within 9 d posttransplant. These preliminary data demonstrate the potency and feasibility of transplantation with highly purified CD34^+^CD90^+^CD45RA^−^ HSCs in the allogeneic setting, providing an option to combine allo-HCT with HSC gene therapy/editing.

## MATERIALS AND METHODS

### Flow Cytometry Analysis and Fluorescence-activated Cell Sorter

Antibodies used for flow-cytometric analysis and fluorescence-activated cell sorting (FACS) of rhesus macaque cells include anti-CD34 (clone 563, BD, Franklin Lakes, NJ), anti-CD45 (clone D058-1283, BD), anti-CD45RA (clone 5H9, BD), and anti-CD90 (clone 5E10, BD). Antibodies were used according to the manufacturer recommendation. Dead cells and debris were excluded via forward scatter/side scatter gating. Flow-cytometric analyses were performed on an Symphony I and FACSAria IIu (BD). Cells for in vitro assays, as well as NHP stem cell transplants, were sorted using a FACSAria IIu cell sorter (BD), and purity was assessed by recovery of sorted cells. CD34^+^CD90^+^CD45RA^−^ sorting for transplantation was performed in yield mode to increase cell recovery, whereas cells for colony-forming cell (CFC) assays were sorted in purity mode to prevent crosscontamination by different subsets.

### Colony-forming Cell Assay

For CFC assays, 1000 to 1200 sorted cells were seeded into 3.5 mL ColonyGEL 1402 (ReachBio, Seattle, WA). Hematopoietic colonies were scored after 12 to 14 d of culture. Arising colonies were identified as colony-forming unit- (CFU-) granulocyte (CFU-G), macrophage (CFU-M), granulocyte-macrophage (CFU-GM), and burst-forming unit erythrocyte. Colonies consisting of erythroid and myeloid cells were scored as CFU-MIX.

### NHP Animal Housing and Care/Ethics Statement

Healthy juvenile rhesus macaques were housed at the University of Washington National Primate Research Center (WaNPRC) under conditions approved by the American Association for the Accreditation of Laboratory Animal Care. All experimental procedures performed were reviewed and approved by the Institutional Animal Care and Use Committee of the Fred Hutchinson Cancer Research Center and University of Washington (Protocol no. 3235-06). This study was performed in strict accordance with the recommendations in the Guide for the Care and Use of Laboratory Animals of the National Institutes of Health (they were HLA-typed and assigned based on their genotype as described in the results section. No random assignment). This study included at least twice daily observation by animal technicians for basic husbandry parameters (eg, food intake, activity, stool consistency, and overall appearance) as well as daily observation by a veterinary technician and/or veterinarian. Animals were housed in cages approved by The Guide and in accordance with Animal Welfare Act regulations. Animals were fed twice daily and were fasted for up to 14 h before sedation. If a clinical abnormality was noted by the WaNPRC personnel, standard WaNPRC procedures were followed to notify the veterinary staff for evaluation and determination for admission as a clinical case. Animals were sedated by administration of ketamine HCl and telazol and supportive agents before all procedures. Following sedation, animals were monitored according to WaNPRC standard protocols. Analgesics were provided as prescribed by the Clinical Veterinary staff for at least 48 h after the procedures and could be extended at the discretion of the veterinarians, based on clinical observations. Decisions to euthanize animals were made in close consultation with veterinary staff and were performed in accordance with guidelines as established by the American Veterinary Medical Association Panel on Euthanasia (2013). Prior to euthanasia, animals were sedated by administration of ketamine HCl.

### Stem Cell Isolation and Allogeneic NHP Transplantation

Allogeneic NHP transplants, priming (mobilization), collection of cells, and sort-purification of the CD34^+^CD90^+^CD45RA^−^ subset were conducted consistent with our previously published protocols.^26,29,30^ Briefly, 2 male animals were pretreated with granulocyte colony-stimulation factor (G-CSF) (50 μg/kg) and SCF (50 μg/kg) for 4 d to prime the BM. On day 4, BM aspirates were performed, and CD34^+^ cells were isolated by immunomagnetic cell separation with the aid of an anti-CD34 antibody (clone12.8) and anti-IgM microbeads (magnetic-assisted cell sorting technology [MACS]; Miltenyi Biotech). CD34^+^CD90^+^CD45RA^−^ cells were sort-purified and pulsed for 2 h in PBS supplemented with 10 µmol/L PGE-2 (Cayman Chemical Company, Ann Arbor, MI) to promote engraftment before infusion as previously described.^31,32^ One day before and on the day of donor cell infusion, recipient macaques were conditioned with myeloablative total body irradiation (1020 cGy) from a 6 MV x-ray beam of a single-source linear accelerator (Varian Clinac 23EX Energy Linear Accelerator) at the Fred Hutchinson Cancer Research Center (Seattle, Washington). Irradiation was administered in 4 equal doses over 2 d at a rate of 7 cGy/min as previously described^33^ before cell infusion.

**FIGURE 1.**
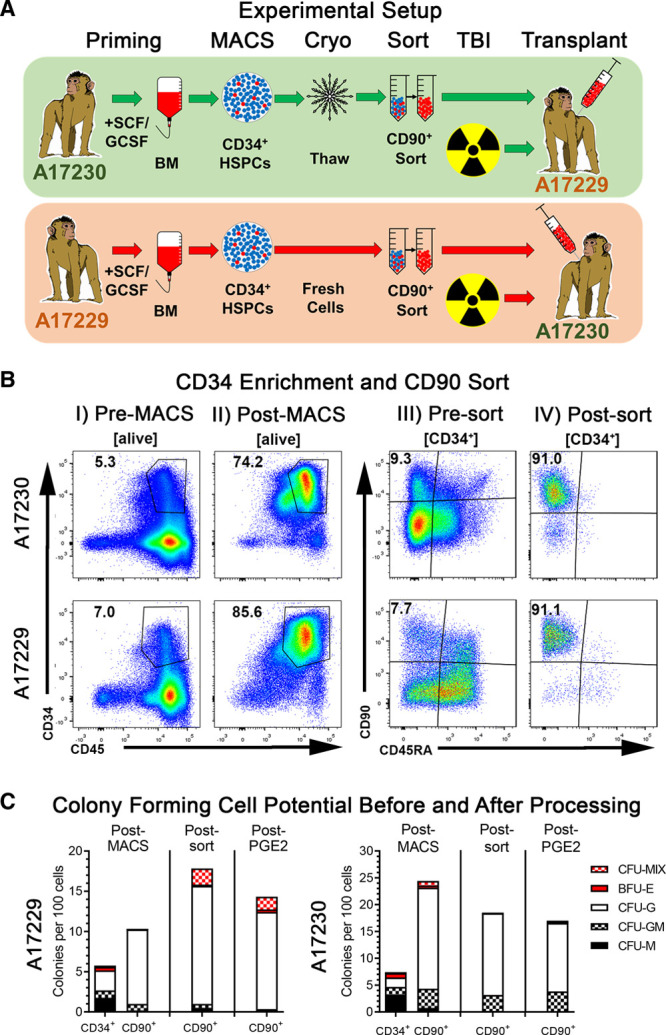
Transplant scheme and pretransplant stem and progenitor cells quality control. A, Experimental setup for the allogenic transplantation of sort-purified CD34^+^CD90^+^CD45RA^−^ cells in rhesus macaques. B, Flow-cytometric quality control of CD34^+^ cells pre-MACS and post-MACS as well as CD34^+^CD90^+^CD45RA^−^ cells presort and postsort. C, Colony-forming cell (CFC) potential of CD34^+^ and CD34^+^CD90^+^CD45RA^−^ cells across the different processing steps. BFU-E, burst-forming unit erythrocyte; BM, bone marrow; CFU, colony-forming unit; G, granulocyte; HSPC, hematopoietic stem and progenitor cell; M, monocyte/macrophage; MACS, magnetic-assisted cell sorting; MIX, erythrocytes, granulocytes, and monocytes/macrophages; TBI, total body irradiation.

Because of logistical constrains not permitting 2 transplants simultaneously, animal transplants were offset in time. In the order of events (also shown in Figure 1A), animal ID A17229 was G-CSF treated from April 14, 2018, until April 18, 2018, BM retrieved on April 18, 2018, CD34^+^ cells enriched, and CD34^+^ cells cryopreserved in 5% dimethyl sulfoxide, 95% fetal bovine serum. After white blood cell (WBC) counts in A17229 were back at baseline, animal ID A17230 was G-CSF-treated from May 27, 2018, until May 31, 2018. On May 31, 2018, BM was collected, CD34^+^ cells were enriched, and CD34^+^CD90^+^CD45RA^−^ cells were sort-purified and transplanted into the myeloablative conditioned animal ID A17229. After WBC counts in animal ID A17230 were back at baseline, cryopreserved CD34^+^ from animal ID A17229 were thawed (on September 05, 2018), and CD34^+^CD90^+^CD45RA^−^ cells were sort-purified and transplanted into the myeloablative conditioned animal ID A17230.

Following CD34^+^CD90^+^CD45RA^−^ transplantation, G-CSF (10 μg/kg) was administered daily from the day of cell infusion to support neutrophil recovery. Graft versus host disease/rejection prophylaxis starting the day of cell infusion included tacrolimus alone (0.025 mg/kg twice daily, with a target serum level of 5–10 ng/mL) for the first transplant recipient ID A17230, and tacrolimus combined with sirolimus (target serum level of 5–15 ng/mL) for the second transplant recipient ID A17229 because of the adverse events observed in animal ID A17230. Supportive care, including antibiotics, electrolytes, and fluids, was given as necessary. Blood counts were analyzed daily to monitor hematopoietic recovery; whole blood or platelets-rich plasma transfusions were administered when platelet counts dropped below 50 000/μL. Antiviral, antifungal, and antibacterial prophylaxis included acyclovir (10 mg/kg IV daily), cidofovir (5 mg/kg IV weekly), vancomycin (20 mg/kg daily), ceftazidime (150 mg/kg IV daily), and fluconazole (5 mg/kg orally or IV daily) as previously described.^34^

### MHC Typing

MHC typing was performed by both microsatellite and allele-specific MHC typing.^30^ Transplant donors and recipients were fully MHC-matched, full siblings, and were typed as follows: haplotype 1: A001, B047a, DR04a; haplotype 2: A012, B001a, DR03a.

### Chimerism Analysis

Donor chimerism analysis was performed on whole blood, on total unfractionated BM collected at necropsy, and on colony-forming units obtained following end point BM culture for 12 to 14 d via molecular analysis of divergent microsatellite DNA markers, as previously described.^30,35,36^

## RESULTS

### Efficient Sort-purification of Rhesus Macaque CD90^+^ HSC for Transplantation

Juvenile rhesus macaques were genotyped as previously described,^30^ and 2 male fully MHC-matched, full siblings (animal ID: A17229 and A17230) were selected for this study as illustrated in Figure 1A.

CD34^+^ cells enrichment from A17229 and A17230 BM yielded 80.5 × 10^6^ and 73 × 10^6^ CD34^+^ cells with a purity of 74.2% and 85.6%, respectively (Table 1 and Figure 1B). CD34-enriched cell fractions contained 9.3% (A17229) and 7.7% (A17230) CD34^+^CD90^+^CD45RA^−^ HSPCs, respectively. Sort-purification of CD34^+^CD90^+^CD45RA^−^ cells resulted in 2.4 × 10^6^ (A17229) and 2.05 × 10^6^ (A17230) cells with a purity >90% in both sorts. Animal ID A17229 received 417 000 and animal ID A17230 422 000 CD34^+^CD90^+^CD45RA^−^ cells/kg (Table 1).

**TABLE 1.**
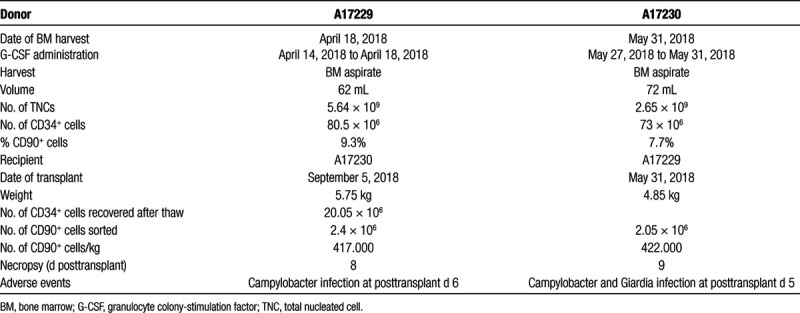
Animal and transplant characteristics

To confirm the maintenance of differentiation potential throughout the selection process, total CD34^+^ HSPCs, as well as CD34^+^CD90^+^CD45RA^−^ HSPC, were introduced into CFC assays after CD34-enrichment (post-MACS), after flow-based cell sorting (postsort), and after PGE-2 pulse (post-PGE-2; Figure 1C). In agreement with our previous studies,^26,28^ sort-purified CD34^+^CD90^+^CD45RA^−^ HSPCs showed a higher frequency of cells with CFC potential in comparison to total CD34^+^ cells, and a characteristic bias towards CFU-G colonies. However, cryopreserved CD34^+^ cells and subsets from animal ID A17229 showed slightly reduced colony-forming potential in comparison to freshly processed cells from animal A17230. Comparison of CD34^+^CD90^+^CD45RA^−^ CFC data at different processing steps demonstrated that CFC frequency and colony composition remained unaffected.

In summary, we successfully purified CD34^+^CD90^+^CD45RA^−^ HSPCs in high numbers and purity by combining CD34 MACS with flow-based CD90 cell sorting. Most importantly, this combined approach did not impact the in vitro differentiation potential of the HSC-enriched CD34 subset providing a viable strategy to generate a high-quality HSPCs for allogeneic transplantation.

### Donor Cell Chimerism in the Recipients’ BM Following Transplantation With Highly Purified CD34^+^CD90^+^CD45RA^−^ Cells

**FIGURE 2.**
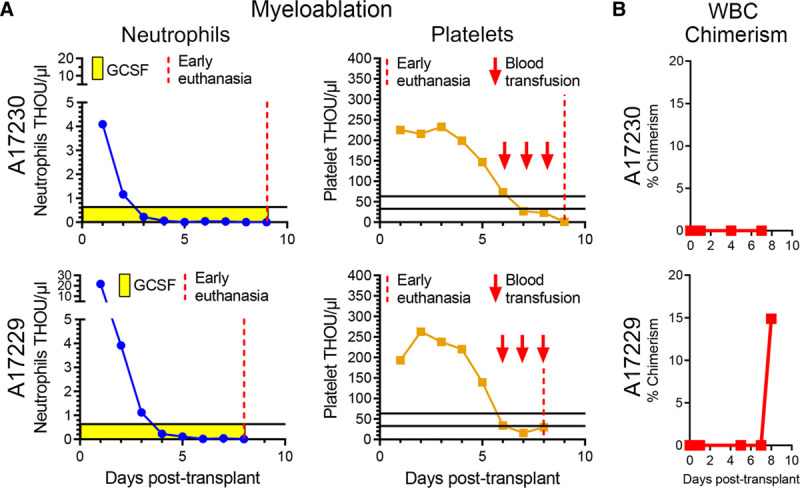
Posttransplant follow-up and chimerism analysis in peripheral blood (PB). A, Neutrophil and platelet counts posttransplant until early euthanasia (vertical red dashed line). B, Frequency of donor chimerism in PB white blood cells (WBCs) determined by microsatellite DNA analysis.

Neutrophil and platelet counts were monitored daily posttransplantation (Figure 2A). Neutrophil counts dropped below 500 cells per µL 3 to 4 d following total body irradiation and remained almost undetectable for the entire experimental follow-up, as the experiments were terminated early because of infectious complications, specifically at day 8 posttransplant for A17229 and at day 9 for A17230. Animal ID A17229 was found to be positive for campylobacter at day 6 posttransplant, which resulted in persistent thrombocytopenia despite daily blood support, bloody stool, and ultimately severe multifocal gastrointestinal and brain hemorrhages. Animal ID A17230 was found to be both campylobacter and giardia positive at day 5 posttransplant, and euthanasia was elected because of severe thrombocytopenia, acutely elevated C-reactive protein, and blood urea nitrogen, as well as clinical disorientation. A17230 platelet counts were stable for the first 3 d and rapidly decreased below the threshold of 50 000 per µL at day 6 to 7 posttransplant and remained below 20 000 per µL despite daily transfusions starting at day 6 posttransplant, likely because of internal gastrointestinal bleeding following campylobacter and giardia infection.

PB samples were taken at multiple time points posttransplant and analyzed for donor chimerism (Figure 2B). Although we were unable to detect donor PB chimerism in animal A17230 before necropsy, we observed a sharp increase in PB donor chimerism in animal ID A17229 at day 8 posttransplant, with about 15% of peripheral WBCs being of donor.

**FIGURE 3.**
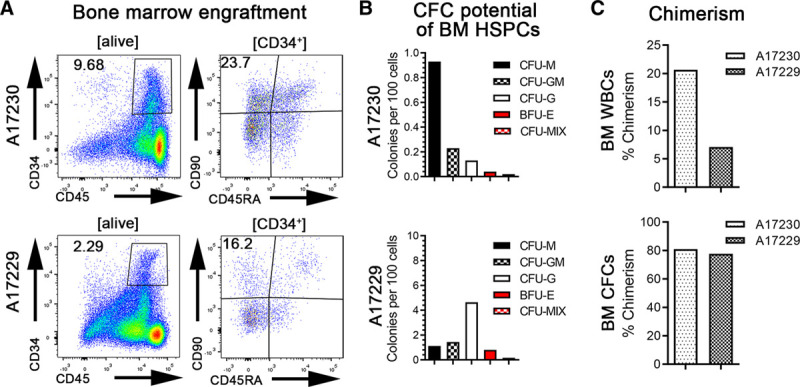
Bone marrow (BM) engraftment and chimerism at necropsy. A, Flow-cytometric assessment of total CD34^+^ cells as well as CD34^+^CD90^+^CD45RA^−^ cells in the BM at the d of necropsy. B, Colony-forming cell (CFC) potential of different numbers of BM white blood cells (WBCs) cells at the d of necropsy. C, Donor chimerism in total BM WBCs and BM CFCs at the d of necropsy (d 8 and 9 for A17229 and A17230, respectively) determined by microsatellite DNA analysis. BFU-E, burst-forming unit erythrocyte; CFU, colony-forming unit; G, granulocyte; HSPC, hematopoietic stem and progenitor cell; M, monocyte/macrophage; MIX, erythrocytes, granulocytes, and monocytes/macrophages.

At necropsy, we isolated BM for engraftment and chimerism analysis (Figure 3). Flow-cytometric assessment revealed that the BM niche contained high frequencies of CD34^+^ subsets, including HSC-enriched CD34^+^CD90^+^CD45RA^−^, multipotent and erythro-myeloid progenitors-enriched CD34^+^CD90^−^CD45RA^−^, and lympho-myeloid progenitor-enriched CD34^+^CD90^−^CD45RA^+^ HSPCs (Figure 3A). Bulk BM WBCs were plated into CFC assays to confirm engraftment of CD34^+^ HSPCs with multilineage differentiation potential (Figure 3B). In both animals, BM resident CD34^+^ HSPCs gave rise to myeloid, erythroid, as well as erythro-myeloid, colonies. Chimerism analysis revealed that donor chimerism reached 20% in animal A17230 and 7% in A17229 in whole BM, whereas analysis of BM-derived, cultured CFCs demonstrated ≈80% donor chimerism for both animals (Figure 3C).

In summary, sort-purified CD34^+^CD90^+^CD45RA^−^ cells successfully homed and rapidly engrafted in both hosts. Significant early donor chimerism was observed in the BM compartment, followed by initial onset of donor chimerism in the PB as early as 9 d posttransplant in 1 of the 2 transplanted animals.

## DISCUSSION

Here, we report the results of the first proof of concept allogeneic transplantation study with highly purified HSC-enriched CD34^+^CD90^+^CD45RA^−^ cells in an NHP model. We successfully transplanted low numbers of sort-purified CD34^+^CD90^+^CD45RA^−^ cells into myeloablative conditioned hosts and observed early engraftment of donor cells in the BM stem cell compartment, as well as initial onset of PB chimerism as early as 8 d posttransplant in the absence of cotransplanted accessory cells. This study demonstrates that (1) the large-scale purification of an HSC-enriched CD34^+^ subset is technically feasible, (2) that sorted cells retain their capability to engraft in the BM, and (3) that the transplantation of very low numbers of a unique HSC-enriched cell fraction can lead to rapid BM and peripheral donor chimerism following allogeneic transplantation.

Engraftment and reconstitution of the hematopoietic system after myeloablative conditioning are believed to occur in waves initially thought to be driven by more committed multipotent progenitor cells and later on by long-term engrafting HSCs, all of which are all contained in the total CD34^+^ cell fraction.^37–39^ By performing competitive repopulation experiments of phenotypically defined CD34^+^ subsets in the preclinical NHP stem cell transplantation model, we have previously shown that the CD34^+^CD90^+^CD45RA^−^ fraction of total CD34^+^ HSPCs was exclusively responsible for rapid short-term as well as robust long-term multilineage reconstitution.^26^ Similarly, we recently reported that selective genetic modification of the CD34^+^CD90^+^CD45RA^−^ HSPC subset alone was required in order to reach high-level and sustained engraftment of gene-edited cells in an autologous NHP transplantation model.^28^ Although both of these studies were performed by cotransplanting sort-purified, gene-modified CD34^+^CD90^+^CD45RA^−^ cells with unmodified CD34^+^CD90^−^ HSPCs, we here show that the transplantation of low numbers (417 000–422 000 cells per kg) of unmodified, highly purified CD34^+^CD90^+^CD45RA^−^ cells alone is sufficient to obtain early BM donor chimerism, as well as the onset of donor chimerism in the periphery following allogeneic transplantation. Thus, similarly to the results obtained in our xenograft model using NHP-derived HSPCs transplanted into MISTRG mice,^27,40^ cotransplantation of CD34^+^CD90^−^ progenitor cells was not required for the engraftment of the CD34^+^CD90^+^CD45RA^−^ subset in the allogeneic setting.

CFC assays performed by culturing BM cells obtained at necropsy demonstrated that purified and engrafted CD34^+^CD90^+^CD45RA^−^ cells gave rise to all downstream hematopoietic progenitor cells within only 8 to 9 d posttransplant. Most importantly, the vast majority of engrafted CD34^+^ cells with erythro-myeloid CFC potential were host-derived, indicating successful homing of primitive HSPCs, maintenance of differentiation potential following cell sorting, and capacity to rapidly recover host hematopoiesis. In 1 of the 2 recipients studied, we even observed a rapid increase of chimerism in the PB at day 8 posttransplant, indicating that engrafted CD34^+^CD90^+^CD45RA^−^ cells are capable to fully mature and reach the periphery shortly after transplant.

One objective of the study was to determine exclusive engraftment of CD90 positive cells as a possible means to further deplete T cells and T-cell progenitors in the graft. However, these experiments had to be terminated early because of infectious complications, consistent with the high risk of infection after allo-HCT with T-cell–depleted grafts. Future studies are needed to assess the feasibility of this approach transplanting greater numbers of CD90^+^ cells per kg body weight to shorten the time to neutrophil recovery, implement improved antimicrobial coverage as well as upfront screening, and test the potential add-back of mature T cells or other progenitor cells to improve protective immunity posttransplant.

A second major objective was to determine whether the CD90 population could contribute to engraftment in the allogeneic setting for gene editing. Follow-up studies will be needed to combine our established allo-HCT^33^ and gene therapy protocols,^26,28^ comprehensively evaluate the engraftment potential of sort-purified and gene-modified CD90^+^ HSPCs, and further evaluate the necessity of an T cell add-back during combining both procedures.

Initial clinical attempts that aimed to enrich for a phenotypically defined, HSC-enriched CD34^+^ subpopulation for autologous transplantation in humans were performed in the 1990s.^41–43^ Flow-based cell sorting HSC-enrichment strategies focused on lin^−^CD34^+^CD90^+^ or CD34^+^CD90^+^ cell fractions that were phenotypically depleted of malignant cells and were evaluated in autologous stem cell transplants for the treatment of multiple myeloma, breast cancer, and non-Hodgkin lymphoma.^41–43^ Rapid and sustained engraftment were seen in patients affected by multiple myeloma and breast cancer. These initial studies show that the purification of HSC-enriched CD34-subpopulations for autologous and very likely allogeneic transplantation is technically feasible in humans. Furthermore, transplantation with T-cell depleted, magnetically purified total CD34^+^ HSPCs was recently shown to engraft in the absence of grade 3 to 4 graft versus host disease in 45 patients with Fanconi Anemia following radiation-free reduced intensity conditioning including low dose busulfan, cyclophosphamide, fludarabine, and rabbit antithymocyte globulin.^44^ Our study sets the groundwork for a further development of allogeneic transplantation strategies using purified HSC-enriched CD34^+^CD90^+^CD45RA^−^ cells in the context of both myeloablative and reduced intensity conditioning regimens, in settings where gene-modification of the allo-HCT is required.

As noted above, the ability to sort-purify a genetically modifiable, fully functional, HSC-enriched CD34 subset in the absence of more committed progenitors and cotransferred T cells could enable the possibility to combine allogeneic transplantation with HSC-mediated gene therapy. Treatment strategies that would highly benefit from such a combined approach include: (1) the treatment of HIV patients with genetically modified donor HSPCs lacking the CCR5 receptor, which is necessary for viral entry for most primary viral isolates^7,8^; (2) transplantation of donor cells engineered to reactivate the expression of fetal hemoglobin to treat hemoglobinopathies^28^; and (3) transplantation with gene-modified CD33 knockout donor stem cells in order to confer resistance to CD33-targeted immunotherapy in patients with acute myeloid leukemia.^10^ In all these cases and in many other diseases, the purification of functional allogeneic CD34^+^CD90^+^CD45RA^−^ cells would increase the gene targeting efficiency of long-term engrafting multipotent HSCs, reduce the overall costs for modifying reagents (such as lentiviral vectors or CRISPR/Cas9 mRNA/RNPs), and ultimately increase transplant feasibility,^45^ as well as extending the option of allogeneic stem cell transplantation to a growing number of patients.^46^

In summary, we here provide evidence that the purification of functional HSC-enriched CD34^+^CD90^+^CD45RA^−^ cells is feasible in the allogeneic transplantation setting and rapidly leads to the differentiation of all hematopoietic progenitors in the BM as well as in the periphery. This novel approach may lead to the development of next-generation, more selective allogeneic transplantation strategies aimed at selectively delivering target genes of interest to the hematopoietic compartment for the treatment of a wide array of hematologic and nonhematologic disorders.

## ACKNOWLEDGMENTS

We thank Helen Crawford for help in preparing this manuscript. We also thank Veronica Nelson, Michelle Hoffmann, Erica Curry, and Kelvin Sze for excellent support in our rhesus macaque studies.
